# Floating Oscillator-Embedded Triboelectric Generator for Versatile Mechanical Energy Harvesting

**DOI:** 10.1038/srep16409

**Published:** 2015-11-10

**Authors:** Myeong-Lok Seol, Jin-Woo Han, Seung-Bae Jeon, M. Meyyappan, Yang-Kyu Choi

**Affiliations:** 1 Department of Electrical Engineering, Korea Advanced Institute of Science and Technology (KAIST), 291 Daehak-ro, Yuseong-gu, Daejeon 305-701, Republic of Korea; 2 Center for Nanotechnology, NASA Ames Research Center, Moffett Field, California 94035, United States

## Abstract

A versatile vibration energy harvesting platform based on a triboelectricity is proposed and analyzed. External mechanical vibration repeats an oscillating motion of a polymer-coated metal oscillator floating inside a surrounding tube. Continuous sidewall friction at the contact interface of the oscillator induces current between the inner oscillator electrode and the outer tube electrode to convert mechanical vibrations into electrical energy. The floating oscillator-embedded triboelectric generator (FO-TEG) is applicable for both impulse excitation and sinusoidal vibration which universally exist in usual environment. For the impulse excitation, the generated current sustains and slowly decays by the residual oscillation of the floating oscillator. For the sinusoidal vibration, the output energy can be maximized by resonance oscillation. The operating frequency range can be simply optimized with high degree of freedom to satisfy various application requirements. In addition, the excellent immunity against ambient humidity is experimentally demonstrated, which stems from the inherently packaged structure of FO-TEG. The prototype device provides a peak-to-peak open-circuit voltage of 157 V and instantaneous short-circuit current of 4.6 μA, within sub-10 Hz of operating frequency. To visually demonstrate the energy harvesting behavior of FO-TEG, lighting of an array of LEDs is demonstrated using artificial vibration and human running.

With the rapid development of wireless sensor networks and portable electronic devices, the demand for mobile electricity sources has been continuously growing. The conventional power source is based on batteries, but the finite energy capacity and the recharging process are problematic^1^. Harvesting energy from our environment has recently become a promising alternative for sustainable but moderate power source. In this sense, mechanical energy is an attractive source because of its abundance in nature and large energy capacity. Electromagnetic^2^,^3^,^4^, electrostatic^5^,^6^,^7^, and piezoelectric^8^,^9^,^10^,^11^,^12^ energy harvesters have been traditionally used to convert ambient mechanical energy into usable electricity. Electromagnetic energy harvester is based on the current induction by Lenz’s law, electrostatic energy harvester is based on the internal vibration of a movable capacitor, and piezoelectric energy harvester is based on the asymmetric charge distribution of piezoelectric materials. More recently, a triboelectric generator (TEG) which combines contact electrification and electrostatic induction mechanisms was introduced^13^. The TEG iteratively generates repeating electrical energy when mechanical force changes the relative displacement of the triboelectric layer, which contains strong fixed charges on the nanostructured surface. Intensive interest in TEG is attributed to the practical advantages such as high output power^14^,^15^,^16^, design flexibility^17^,^18^,^19^,^20^,^21^,^22^, wide material availability^23^,^24^,^25^, various energy sources^26^,^27^,^28^,^29^, wide application spectrum^30^,^31^,^32^,^33^, and low temperature fabrication^34^,^35^.

Ambient mechanical energy can be categorized into impulse excitation and sinusoidal vibration. Impulse excitations usually present large amplitude with short duration time, while sinusoidal vibrations are mostly found in the form of small amplitudes and high frequency. Both impulse excitations^36^,^37^,^38^,^39^ and sinusoidal vibrations^40^,^41^,^42^,^43^,^44^,^45^,^46^ are widely focused sources of TEG. However, mechanical energy in actual environment are usually not in a single amplitude and frequency wave but in a random and mixed form, which increases design difficulty of a practical mechanical energy harvester. Accordingly, the mechanical energy harvester design needs to be versatile to deal with different types of target mechanical energy sources. In addition, the device size, resonance frequency, and packaging availability should be addressed for practicality.

In this work, a versatile TEG platform based on the floating oscillator is proposed. A polymer-coated metal oscillator is floated inside a hollow tube by the repulsive force of magnets, and the oscillator responds with repeated oscillation when mechanical excitation is applied. The oscillation causes repeated sliding friction between the polymer surface of the inner oscillator and the metal surfaces of the outer tube, which results in triboelectric charging and electrostatic induction. The floating oscillator-embedded triboelectric generator (FO-TEG) is designed to harvest both the impulse excitation and sinusoidal vibration. Arbitrary input mechanical motion is first converted into a linear oscillation of the floating oscillator, which can be effectively converted into the electrical energy. The operating frequency range of the FO-TEG can be flexibly tuned by engineering the structural parameters such as oscillator height and mass. In addition, the triboelectric interface where contact electrification occurs is protected by the encapsulated tube to form a self-packaged structure, which improves immunity against undesired environmental effects such as humidity and debris^36^,^38^,^45^.

## Results and Discussion

### Structure and operation principle

**Figure 1:**
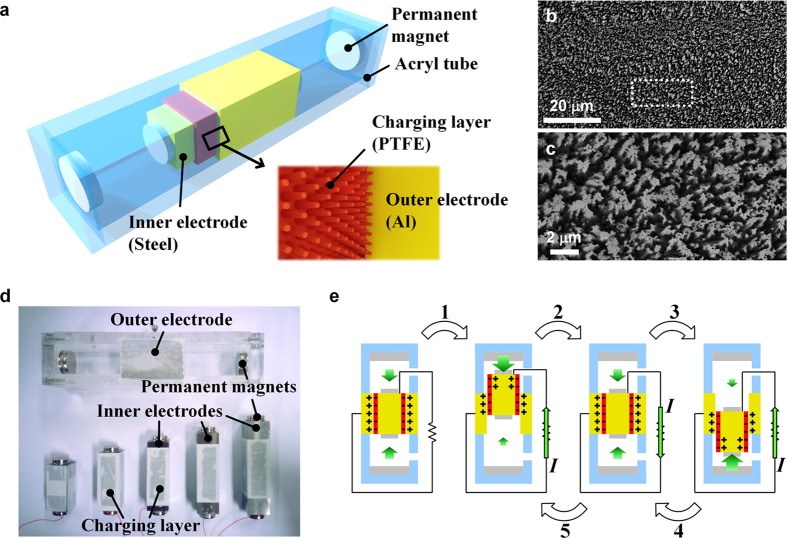
Structure and operation principle of the FO-TEG. (**a**) Schematic illustrating the structure of the FO-TEG. The FO-TEG consists of a tube part with a mobile oscillator part floating inside, suspended by magnetic repulsive forces. Vertical nanowire-like structures are formed on the sidewalls of the PTFE charging layer. (**b**) SEM image of PTFE charging layer. (**c**) Magnified SEM image of the PTFE charging layer. (**d**) Photograph of FO-TEG components. Heights of the oscillator can be flexibly determined to tune the output performance, such as power and resonance frequency. (**e**) Operating principle of the FO-TEG. The position of the oscillator determines the distribution of positive charges on the inner electrode (IE) and outer electrode (OE). Continuous oscillation of the floating oscillator generates consistent output power.

Figure 1a presents a schematic illustration of the FO-TEG. A square pillar oscillator is located inside a square acryl tube. Disk type permanent magnets (NdFeB) are attached at both ends of the tube and the oscillator in the way to face repulsive force. The middle part of the tube is composed of an Al electrode with polished surfaces, which serves as the outer electrode (OE). The OE does not affect the magnetic field inside the tube because of the low permeability of Al (*μ* = 1.26 × 10^−6^ H/m). The oscillator inside the tube is composed of steel (*μ* = 2.52 × 10^−4^ H/m) and serves as the inner electrode (IE). The sidewalls of the IE are coated by 200 μm polytetrafluoroethylene (PTFE) film, which features negative order of electrification and low surface adhesiveness. The PTFE layer serves as the triboelectric layer that contains fixed triboelectric charges. Surface of the PTFE layer is modified by plasma etching to form densely packed nanostructure morphology (Fig. 1b,c). The first role of the nanostructure is to increase the effective surface area. The increased surface area leads to larger triboelectric charge density, which results in enhanced output electrical power. The inner length of the tube excluding the magnet thickness is 13.6 cm and the length of the OE is 5 cm. The heights of the oscillators vary from 5.0 cm to 8.0 cm (Fig. 1d). As oscillator height increases, the neutral airgap distance between tube magnet and oscillator magnet decreases accordingly.

Figure 1e describes the overall operation principle of the FO-TEG. The sidewall contact interface is an important region where contact electrification and electrostatic induction occurs. Due to repeated sliding friction at the interface, the PTFE surface obtains immobile triboelectric charges by the contact electrification process^47^. According to the order of electrification, the PTFE surface obtains negative charges from the Al surface during the repeated contact. The immobile negative triboelectric charges at the PTFE surface act as a charge circulating pump to induce current flow between the IE and the OE. The position of the oscillator determines the distribution of counter positive charges in the IE and the OE. When the oscillator is at a center position, the PTFE surface is entirely overlapped to the OE, so most of the counter positive charges are accumulated in the OE. When the oscillator moves upward by mechanical excitation, a part of the PTFE surface is not overlapped with the OE, so the charge distribution becomes unbalanced (process e-1). To compensate the negative triboelectric charges that are not in contact with the OE, some portion of the positive charges are transferred from the OE to the IE. This charge flow produces the induced current, which is electrical energy to be collected. During the oscillation, the balance of the magnetic repulsive force is also changed because of the distance change between the permanent magnets. The strong magnetic repulsive force between the now closer magnets pushes them apart, so the oscillator starts to move back to the opposite direction. In this process, positive charges flow back from the IE to the OE as the oscillator moves to the center part (process e-2). The oscillator continues moving downward due to the force of inertia, and current flows from OE to IE again for the same reason (process e-3). Ideally, the current is infinitely generated as oscillation recurs (process e-4, e-5). In the real case, the current generation stops due to frictional forces at the sidewall surfaces.

**Figure 2:**
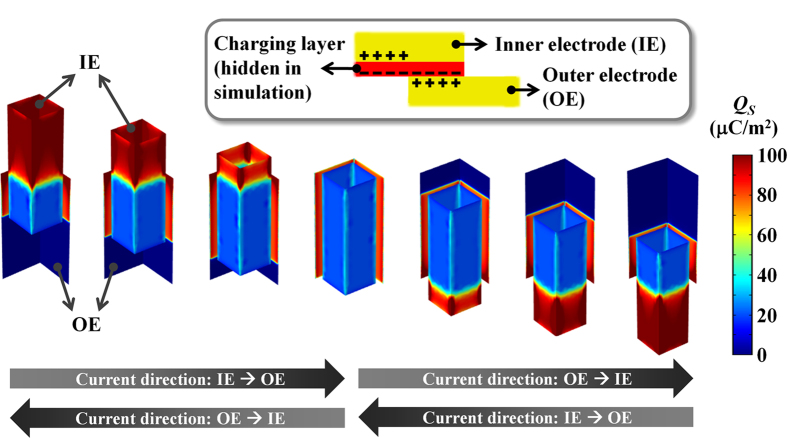
Charge density profiles of inner and outer electrodes. Negative triboelectric charges at the PTFE charging layer attract counter positive charges in the inner electrode (IE) and the outer electrode (OE). Change of the charge density between the IE and the OE with movement of the floating oscillator induces current.

The operating model was confirmed by a finite-element method using COMSOL simulation software (Fig. 2). The selected structural and material parameters follow the actual experimental device. The surface charge density profiles of the IE and the OE corresponding to certain positions of the oscillator are presented as a color map. The PTFE charging layer, which contains a constant negative charge density, is excluded in the color map for better visibility of mobile charges. The triboelectric charge density on the charging layer was assumed to be −100 μC/m^2^. The simulation results are consistent with the conceptually expected profiles. Most of the positive charges are accumulated in the OE when the oscillator is at the center position. The positive charges are transferred from the OE to the IE as the oscillator is separated from the center to upward or downward locations. As expected, a greater amount of flowing charge is observed for larger displacements of the oscillator.

### Modeling for design guideline

**Figure 3:**
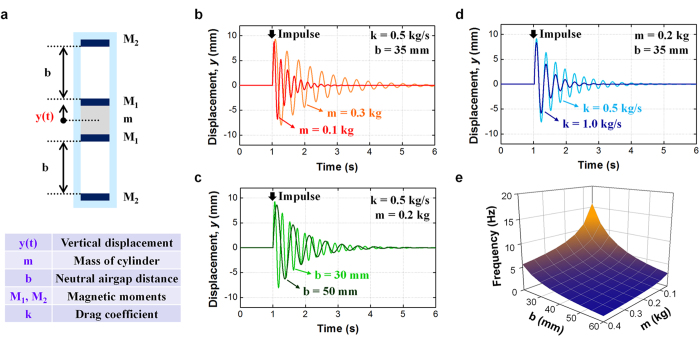
Analytical model of the floating oscillator movement when an impulse is applied. (**a**) Definitions of structural parameters for analytical modeling of the oscillator movement when a sudden impulse is applied to the FO-TEG. (**b**) Damping oscillation model with two different oscillator masses (*m*), which is determined by the oscillator material and size. (**c**) Damping oscillation model with two different drag coefficients (*k*), which is determined by the characteristics of the frictional interface. (**d**) Damping oscillation model with two different neutral airgap distances (*b*), which is determined by the tube length and oscillator height. (**e**) Changing the resonance frequency with various *m* and *b*. Within realistic ranges of *b* and *m*, the resonance frequency is within sub-10 Hz.

Based on the operating principle, oscillations with large amplitude and slower damping are preferred to maximize the induced current. The qualitative design guideline for the FO-TEG can be determined using a simple analytical model. When parameters are defined as shown in Fig. 3a, the relationship between vertical displacement (*y*) and time (*t*) can be modeled by the equation given below. A single delta-function type impulse is assumed as the input mechanical excitation. The detailed modeling process is described in the Supporting information (Figure S1).


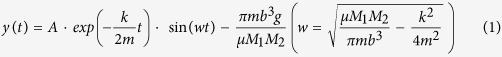


Definitions of variables in the equation are presented in Fig. 3a. The equation presents a sinusoidal wave with exponential damping. The amplitude of the oscillation (*A*) is determined by the amplitude of the applied impulse, and *A* is bounded by the length of the inner oscillator and tube height. This model is valid when *A* is lower than *b,* which is acceptable for most actual environments. The oscillation frequency is a complex function of material parameters and structural parameters. The damping constant is determined by the ratio of the oscillator mass and the drag coefficient. The vertical offset owing to gravitational force is considered when the FO-TEG oscillates in the vertical direction and does not affect the time-dependent movement of the oscillator.

Influence of various parameters on modeled oscillation behaviors are plotted in Fig. 3b–d. Vertical offset caused by the gravitational effect is excluded for easier comparison of relative movements. The results show that the mass of the oscillator (*m*) affects both oscillation frequency and damping ratio (Fig. 3b). A heavy oscillator results in low oscillation frequency and mitigated damping. Considering the range of masses of practically available materials, a large *m* metal such as steel is preferred to maximize the total travel distance of the oscillator. In terms of neutral airgap distance (*b*), which can be controlled by the oscillator height, a small airgap results in faster oscillation at the same damping ratio and thus longer total travel distance (Fig. 3c). A small *b* is preferred from this viewpoint, but too small values for *b* would limit the oscillation amplitude because of collisions between the tube magnet and oscillator magnet. Optimization of *b* requires further actual experimental confirmation, which is presented in the Results and discussion section. In terms of drag coefficient (*k*), which governs frictional force, a small drag coefficient results in a lower damping ratio and the same oscillation frequency (Fig. 3d). Apparently, a material with low surface adhesion is preferred. In this regard, the PTFE film was selected as it has excellent anti-adhesion property as well as strongly negative order of electrification^48^. In addition, the inner surface of the OE is polished to reduce the macro-scale friction.

Resonance frequency is a crucial when a sinusoidal vibration is applied. For applications where the vibration frequency is regular, the device should be carefully designed for the resonance frequency of device to match the vibration frequency. Resonance oscillation occurs when the mechanical vibration and oscillation of the oscillator are synchronized. The resonance frequency of the FO-TEG can be flexibly customized by tuning *m* and *b* values (Fig. 3e). An increase of resonance frequency is accomplished by increasing *m* and *b* or vice versa. However, as change of *m* not only affects the resonance frequency but also the damping ratio, control of *b* value is a preferable strategy to control resonance frequency.

### Experimental measurement and analyses

**Figure 4:**
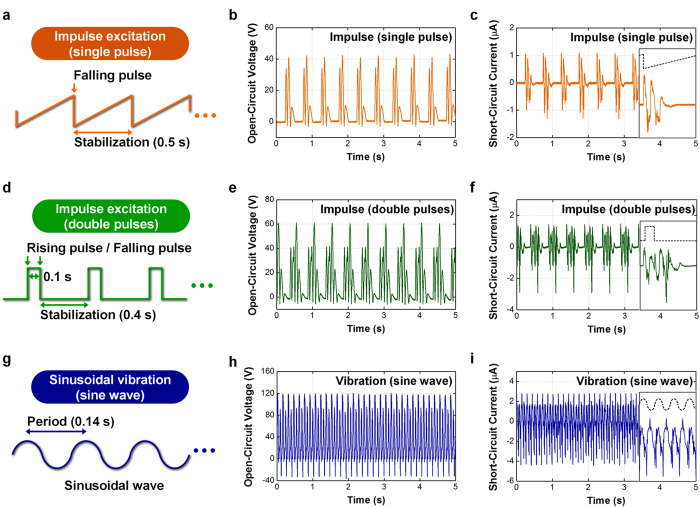
Measurement results of the FO-TEG with various input vibration types. (**a**) A ramp wave applied to an electrodynamic shaker to generate impulse excitation having a single pulse. (**b**) Open-circuit voltage and (**c**) short-circuit current spectrum corresponding to the impulse excitation with single pulse. (**d**) A square wave applied to the electrodynamic shaker to generate impulse excitation with double pulses. (**e**) Open-circuit voltage and (**f**) short-circuit current spectrum corresponding to the impulse excitation with double pulses. (**g**) A sinusoidal wave applied to the electrodynamic shaker to generate sinusoidal vibration. (**h**) Open-circuit voltage and (**i**) short-circuit current spectrum corresponding to the sinusoidal vibration.

Measurement of the FO-TEG was conducted by applying three different mechanical waves: ramp wave, square wave, and sinusoidal wave. The detailed measurement setup is described in the Methods section. The ramp wave from the function generator generates an impulse excitation with a single pulse (Fig. 4a). The single pulse impulse excitations are often found in stepping, hitting, and stopping motions. At the moment of impulse, the oscillator starts moving and electrical energy is generated (Fig. 4b,c). The output current spectrum presents a clear damping oscillation as theoretically predicted. Even though the impulse time was extremely short, oscillation continued for more than 0.25 s after the moment of impulse, which results in extended charge collection time. Compared to conventional contact-separation mode TEGs which are operated by impulse excitation, the FO-TEG provides extended charge collection time. From a practical viewpoint, this output spectrum is advantageous for an energy managing circuit, which is essential for the actual utilization of an energy harvester^49^.

The square wave from the function generator generates an impulse excitation with double pulses (Fig. 4d). The double pulses impulse excitations are found in bouncing, jumping, and repeated impact motions. In this case, the second impulse was applied before the stabilization of damping oscillation caused by the first impulse. From the measurement, two damping oscillations were overlapped and a complicated output spectrum is produced (Fig. 4e,f). Instantaneous voltage and oscillation time increase from 40 V to 60 V and from 0.25 s to 0.45 s, respectively. By integrating the absolute current values, the total induced charge per one mechanical impulse can be calculated. From the calculation, the overlapped oscillation produces even larger current than twice the single damping oscillation, which is attributed to the enhanced oscillation amplitude and the extended oscillation time. Detailed calculation procedure is described in the Supporting information (Figure S2). This result implies that the FO-TEG can also accommodate irregular impulses in actual application environments.

The sine wave from the function generator generates a sinusoidal vibration of the electrodynamic shaker (Fig. 4e). Sinusoidal vibrations are widely found in running, shaking, water waves, and machinery operations. At the resonance frequency, which is determined by the device design, the experimental FO-TEG produced a peak-to-peak open-circuit voltage of 157 V and instantaneous short-circuit current of 4.6 μA (Fig. 4h,i). When the *b* is 4.3 cm and applied frequency is 5.0 Hz, the maximum power is found to be 0.18 mW at load resistance of 50 MΩ. Detailed data about the load resistance dependence are included in the Supporting information (Figure S3). It is attractive that the output power is consistently generated unless the mechanical excitation disappears. This consistent power generation is advantageous for both energy storage applications and devices that require self-powered source.

Influence of vibration amplitude on output open-circuit voltage is analyzed. Peak acceleration is used to represent the input vibration amplitude. Since the peak acceleration is linearly proportional to the input force, increase of the acceleration means increase of the input force. When the *b* value is 4.3 cm, sudden voltage increase is observed at the peak acceleration of 22 m/s^2^. When the peak acceleration is below 22 m/s^2^, the oscillator presents soft fluttering behavior and provides low output voltage. As the peak acceleration exceeds 22 m/s^2^, the oscillator presents resonance oscillation and provides much enhanced output voltage. This means that the input force above certain threshold value is required to induce the resonance oscillation even with the fixed vibration frequency. Qualitatively, when the input force is small, the oscillator cannot overcome the sidewall frictional force and moves the same direction with the surrounding tube. When the input force is large enough, the oscillator moves opposite direction to the tube, so relative displacement is maximized to induce the resonance oscillation. Therefore, the threshold input force to cause resonance can be engineered by tuning oscillator mass, magnetic moment, and frictional force.

**Figure 5:**
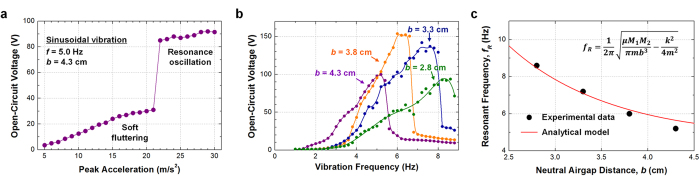
Dependence on vibration amplitude and frequency. (**a**) Output open-circuit voltage when various peak accelerations are applied. The peak acceleration corresponds to the amplitude of input force. Input force should be higher than a certain threshold value to induce the resonance oscillation. (**b**) Output open-circuit voltage with various vibration frequencies. Increase of neutral airgap distance (***b***) increases the operational frequency range. (**c**) Relationship between resonance frequency and *b* value. Experimental resonance frequency is well-matched with the theoretically expected trend. Resonance frequencies are within sub-10 Hz range, which matches to the range that can be found in common outer environments.

A mechanical energy harvester is hard to be practical if the device is only compatible with a specific resonance frequency and cannot be operated at other frequencies. Vibrations in nature are composed of complicatedly overlapped spectra with a wide range of frequencies, generally within the sub-10 Hz range. To address the frequency issue, the output performance of the FO-TEG is investigated with various vibration frequencies. Because the oscillation frequency is not critical parameter for the impulse excitations, only the sinusoidal vibration is focused throughout the frequency experiment. As previously discussed, the resonance frequency of the FO-TEG can be tuned by changing the neutral airgap distance, *b*. The output peak-to-peak open-circuit voltage versus oscillation frequency is measured using various oscillator heights. Embedded oscillators are manually replaced to change b from 2.8 cm to 4.3 cm with a step length of 0.5 cm (Fig. 5a). Oscillation is not initiated in a frequency range less than 2 Hz. During this gentle sinusoidal movement, the oscillator and the tube move together, thus no relative displacement is made. Sliding friction begins when the oscillation frequency exceeds 2 Hz. The output voltage sensitively increases and is finally saturated near the maximum value that is the resonance frequency. As frequency increases further, the output voltage suddenly drops because the oscillator cannot respond to the applied vibration. When *b* is too large because of a too short oscillator, the efficiency becomes significantly lower, because the magnetic repulsive force is too low to overcome the sidewall frictional force. For actual applications, a user should design the *b* value prior to implementation of the FO-TEG to a specific target system in consideration of the predicted frequency range. For example, further increase of *b* is essential to optimize the FO-TEG for sub-3 Hz sinusoidal vibrations, such as water waves.

The experimental frequency dependence is compared with the analytical model (Fig. 5b). Larger *b* values result in a small resonance frequency, and the trend is well-matched with the analytical model. When *b* is decreased from 3.8 cm to 2.8 cm, the power at resonance is decreased 39%, while full width at half maximum (FWHM) is increased 52%. This implies a trade-off between the amount of power at resonance and the available frequency range. Distribution of vibration frequency at a specific application environment should be considered to determine the *b* value. Apparently, the frequency characteristics can be further engineered by modifying other design parameters such as the tube length, oscillator mass, and magnet moments.

**Figure 6:**
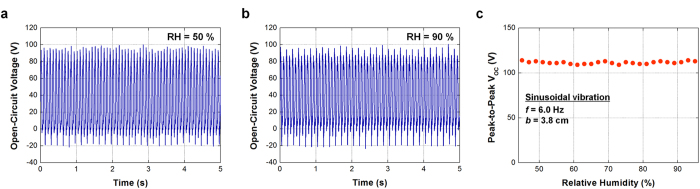
Influence of humidity on output voltage. (**a**) Output voltage spectrum when relative humidity (RH) is 50%. (**b**) Output voltage spectrum when RH is 90%. (**c**) Peak-to-peak voltage values under various RH values. The FO-TEG is independent of humidity in ambient due to the inherently packaged structure.

One distinguished advantage of the FO-TEG is the inherently packaged structure which can improve immunity against the effects from outer environment. In particular, humidity is an important concern in the TEG because the triboelectric charging process is strongly dependent on the humidity^22^,^47^,^50^. From one theory, thin water film is formed on a triboelectric charging layer under a humid environment and the moisture shortens a sustaining time of triboelectric charges. Therefore, output performance of a conventional TEG tends to degrade as the ambient humidity increases. To verify the influence of humidity on the performance of the FO-TEG, electrical measurements are conducted inside a closed chamber, whose relative humidity (RH) is controlled by a humidifier and monitored by a humidity sensor. From the result, the output voltage remains in a constant level under RH ranges from 45% to 95% (Fig. 6a–c). The triboelectric charging layer (i.e. PTFE surface) is protected by the inherently packaged structure, so the triboelectric charge density remains constant regardless of the humidity in ambient. It should be noted that that the unchanged performance even under extremely high humidity (RH = 95%) is firstly demonstrated in this work (Video S1). The immunity against humidity is practically advantageous for consistent energy harvesting in outdoor environment.

**Figure 7:**
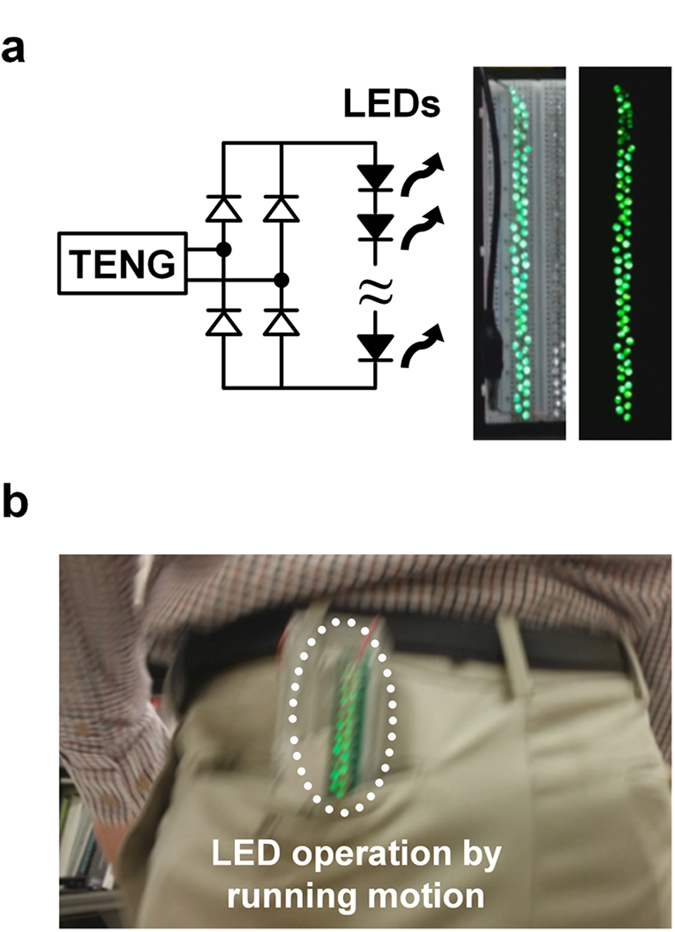
Direct brightening of LEDs with the FO-TEG operation. (**a**) Schematic of a circuit for real-time LED operation and snapshots during the LED operation. 60 LEDs are directly and consistently turned on during continuous vibration. (**b**) Snapshots showing application of the FO-TEG to running motion. To visually confirm power generation, the LEDs are externally connected between two electrodes of the FO-TEG.

To visually confirm the energy generation behavior, array consisting of sixty LEDs were connected in series to the FO-TEG with a full-bridge rectifier circuit (Fig. 7a). Those LEDs consistently brightened during vibration (Video S2). For lightening the serially connected LEDs, the output voltage repeats instantaneous charging and exponential discharging between 115 V to 136 V, and output current fluctuates with the maximum value of 3.7 μA. The output frequency of the spectra corresponds to the cycling frequency. Not only artificial vibration from the electrodynamic shaker but also the naturally generated mechanical motion produced while running turned on the LEDs (Fig. 7b). The running motion, which is composed of a complicated superposition of impulse excitations and sinusoidal vibrations, is harvested by the FO-TEG (Video S3).

## Conclusion

In this report, a floating oscillator-embedded triboelectric generator (FO-TEG) was introduced. Linear oscillation of the floating oscillator induces continuous electrical energy by utilizing the sliding friction at the sidewall contact interface. The FO-TEG has multifaceted applicability due to ambidextrous features: utilization of impulse excitation and continuous sinusoidal vibration. Impulse excitation induces a transient damping oscillation to produce extended energy generating time. This power spectrum is advantageous for the energy management. Continuous sinusoidal vibration induces a resonance oscillation to provide a maximized energy generation, as long as the input frequency is within the operating frequency range. Overall operating frequency range is in a range of sub-10 Hz, which is widely found in natural mechanical energy sources. By simply tuning structural parameters, resonance frequency of the FO-TEG can be fitted to a target application system with a high degree of freedom. To further verify the system level applicability, direct operation of the FO-TEG was demonstrated with human running motion that is a mixed form of impulse excitations and continuous vibrations. With multi-purpose customization based on the selected application area, the FO-TEG is expected to effectively harvest general mechanical energy from the environment, such as human motion, vehicle operation, water waves, machinery-induced vibrations and others.

## Methods

### Nanostructure formation on PTFE surface

Fabrication of the vertical nanowire-like structure on the PTFE film was based on the one-step plasma reactive ion etching process^51^. First, a thin Au layer (5 nm) was deposited using thermal evaporation on a flat polytetrafluoroethylene (PTFE) film (200 μm thickness). Under this condition, Au seeds cannot connect to each other but form an island-like pattern, which can serve as an etching mask pattern. The Au deposited PTFE film was then exposed to a plasma beam. A gas mixture composed of Ar, O_2_, and CF_4_ was released into the chamber with flow rates of 15, 10, and 4 sccm, respectively. The gas pressure was maintained at 10 mTorr during the etching process. A power source of 400 W was used to activate the plasma, and then, 100 W was subsequently applied to accelerate the plasma ions. After 5 min of etching time, the PTFE nanowire-like structure was fabricated.

### Measurement setup

The measurement setup consists of a vibration generating part and an electrical measurement part. First, a function generator (33120, hp) generated and transported an electric signal to a power amplifier (pa-141, Labworks), which was subsequently connected to an electrodynamic shaker (LW-140-110, Labworks). Three wave types were used for the experiment: ramp wave, square wave, and sinusoidal wave. The electrodynamic shaker converted the electric signals into the corresponding vibrations of a rigid oscillator. Output signals generated by the FO-TEG were measured by electrometer (Keithley 6514). Open-circuit voltage (*R*_*load*_ = 200 TΩ) and short-circuit current (*R*_*load*_ = 2 Ω) were measured with a sampling frequency of 5 kHz.

## Additional Information

**How to cite this article**: Seol, M.-L. *et al.* Floating Oscillator-Embedded Triboelectric Generator for Versatile Mechanical Energy Harvesting. *Sci. Rep.* **5**, 16409; doi: 10.1038/srep16409 (2015).

## Supplementary Material

Supplementary Information

Supplementary Videos S1

Supplementary Videos S2

Supplementary Videos S3
