# Supplementary material for: Floating Oscillator-Embedded Triboelectric Generator for Versatile Mechanical Energy Harvesting
Source: Sci Rep. 2015 Nov 10;5:16409. doi: 10.1038/srep16409 (PMC4639750; doi:10.1038/srep16409)
Supplement: Supplementary Information [file srep16409-s1.pdf]

Supporting Information

Floating Oscillator-Embedded Triboelectric Generator for Versatile Mechanical Energy Harvesting

Myeong-Lok Seol[1], Jin-Woo Han[2], Seung-Bae Jeon[1], M. Meyyappan[2], and Yang-Kyu Choi[1,*]

[1] Department of Electrical Engineering, Korea Advanced Institute of Science and Technology (KAIST), 291 Daehak-ro, Yuseong-gu, Daejeon 305-701, Republic of Korea

[2] Center for Nanotechnology, NASA Ames Research Center, Moffett Field, California 94035, United States

* Address correspondence to ykchoi@ee.kaist.ac.kr

Modeling of Oscillation

The purpose of the modeling is to find the relationship for the vertical displacement of the oscillator with time when an instantaneous mechanical impulse is applied. The definitions of the parameters used in the model are described in Figure S1.

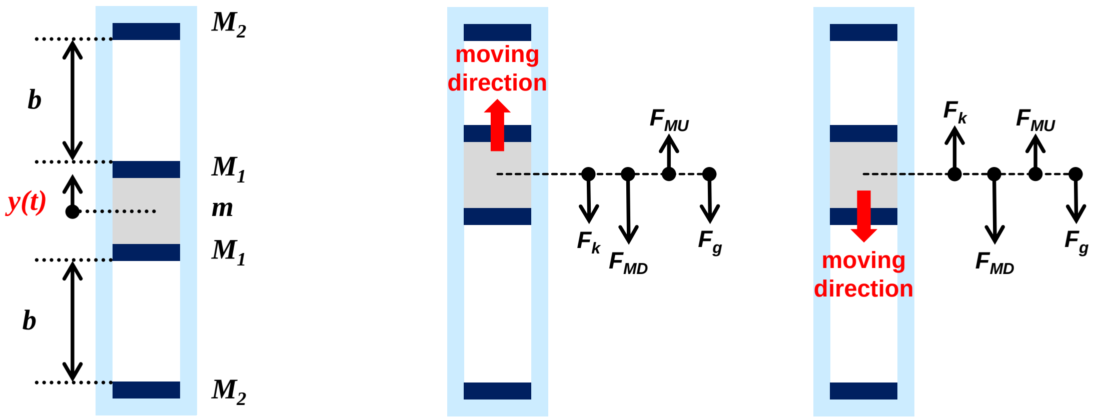

y(t)	**Vertical displacement**
m	**Mass of cylinder**
b	**Neutral airgap distance**
M_1, M_2	**Magnetic moments**
k	**Drag coefficient**

$$F_{MD} = -\frac{\mu M_1 M_2}{4\pi}\frac{1}{(b-y)^2} \qquad F_{MU} = \frac{\mu M_1 M_2}{4\pi}\frac{1}{(b+y)^2}$$

$$F_k = -k\dot{y} \qquad F_g = -mg$$

Figure S1. Definitions of parameters and expressions of forces of the model

The force balance equation at any time and position can be described as follows. The total equivalent force is determined from the combination of upward magnetic force (F_{MU}), downward magnetic force (F_{MD}), drag force (F_k), and gravitational force (F_g).

$$m\ddot{y} = \frac{\mu M_1 M_2}{4\pi}\left\{\frac{1}{(b+y)^2} - \frac{1}{(b-y)^2}\right\} - k\dot{y} - mg \qquad (1)$$

$$m\ddot{y} = \frac{\mu M_1 M_2}{4\pi}\left\{\frac{1}{(b+y)^2} - \frac{1}{(b-y)^2}\right\} - k\dot{y} - mg \tag{2}$$

The equation can be modified using Tayler series.

$$m\ddot{y} = \frac{\mu M_1 M_2}{4\pi b^2}\left\{\left(1 - \frac{y}{b} + \frac{y^2}{b^2} - \frac{y^3}{b^3} + \cdots\right)^2 - \left(1 + \frac{y}{b} + \frac{y^2}{b^2} + \frac{y^3}{b^3} + \cdots\right)^2\right\} - k\dot{y} - mg \tag{3}$$

Assume $b \gg y$ (small oscillation condition), the equation above is simplified as follows.

$$\begin{aligned} m\ddot{y} &= \frac{\mu M_1 M_2}{4\pi b^2}\left\{\left(1 - \frac{2y}{b}\right) - \left(1 + \frac{2y}{b}\right)\right\} - k\dot{y} - mg \\ &= -\frac{\mu M_1 M_2}{\pi b^3} y - k\dot{y} - mg \end{aligned} \tag{4}$$

The linear second-order differential equation can be rewritten as follows.

$$\ddot{y} + \frac{k}{m}\dot{y} + \frac{\mu M_1 M_2}{\pi m b^3} y + g = 0 \tag{5}$$

The analytical solution of the linear second-order differential equation is well-known. At the oscillation condition, *D* shown below contains imaginary part$\left(i.e.,\ k^2 < 4\frac{\mu M_1 M_2 m}{\pi b^3}\right)$.

$$y = A \cdot exp(D_1 t) + B \cdot exp(D_2 t) + C \qquad \left(D = \frac{-k \pm \sqrt{k^2 - 4\frac{\mu M_1 M_2 m}{\pi b^3}}}{2m}\right) \tag{6}$$

Before the impulse is applied at the moment *t*=0, the oscillator is at neutral position that only gravity effect governs the constant vertical offset. Therefore, A, B, and C values can be determined by the boundary condition.

$$y(t = 0) = -\frac{\pi m b^3 g}{\mu M_1 M_2} \tag{7}$$

$$\mathbf{B} = -\mathbf{A}, \quad \mathbf{C} = -\frac{\pi m b^3 g}{\mu M_1 M_2} \tag{8}$$

Then, the finalized equation becomes

$$y = A \cdot exp\left(-\frac{k}{2m}t\right)\{exp(iwt) - exp(-iwt)\} - \frac{\pi m b^3 g}{\mu M_1 M_2} \quad \left(w = \sqrt{\frac{\mu M_1 M_2}{\pi m b^3} - \frac{k^2}{4m^2}}\right) \tag{9}$$

From the Euler's formula, $\mathbf{exp}(ix) = \mathbf{cos}(x) + i\mathbf{sin}(x)$,

$$y = A \cdot exp\left(-\frac{k}{2m}t\right) \cdot \sin(wt) - \frac{\pi m b^3 g}{\mu M_1 M_2} \tag{10}$$

This equation describes sinusoidal wave with constant vertical offset and exponential damping.

Detail Procedure of the Charge Calculation

Figure S2 presents magnified current spectra when single pulse excitation and double pulses excitation are applied, respectively. Since the output current value means transferred charge per unit time, generated charge can be reversely calculated by integral of current values with respect to the total oscillation time. From the calculation result, double pulses induce 0.195 μC per an excitation, which is 2.19 times larger charges compared with the single pulse. Qualitatively, overlap of two damping oscillations induces a large displacement of the oscillator, which cannot be made by a single damping oscillation. As a result, both instantaneous amplitude and total oscillation time are extended.

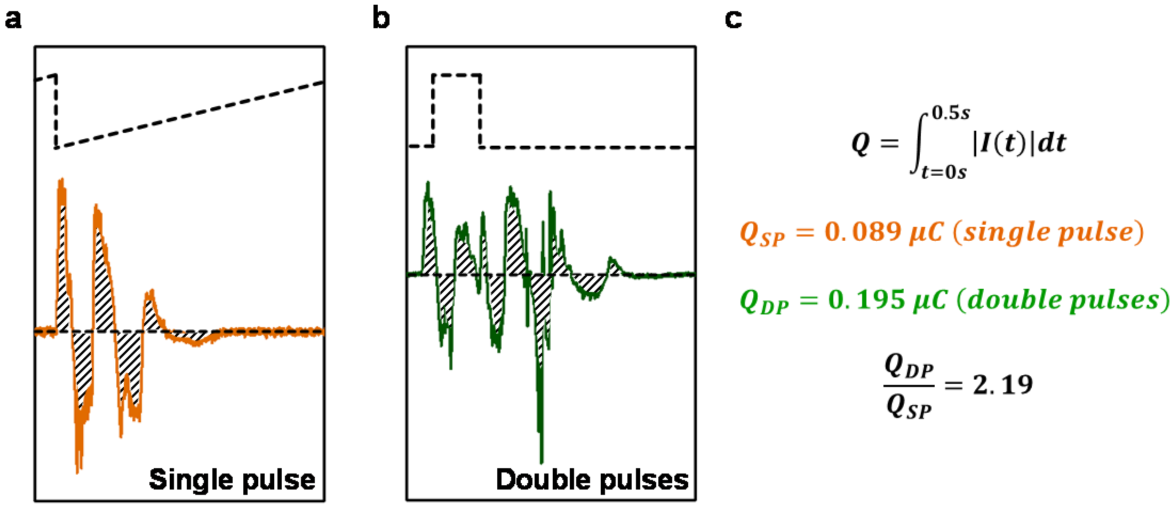

Figure S2. (a) A magnified current spectrum when single pulse excitation is applied. (b) A magnified current spectrum when double pulses excitation is applied. (c) Calculated transferred charges per an excitation.

Load Resistance Dependence

Figure S3 presents output voltage and current of the FO-TEG for various load resistances. The voltage increases and the current decreases as the load resistance increases. Output power is calculated by multiplying the voltage and the current value. The maximum output power is found to be 0.18 mW when the load resistance is 50 MΩ.

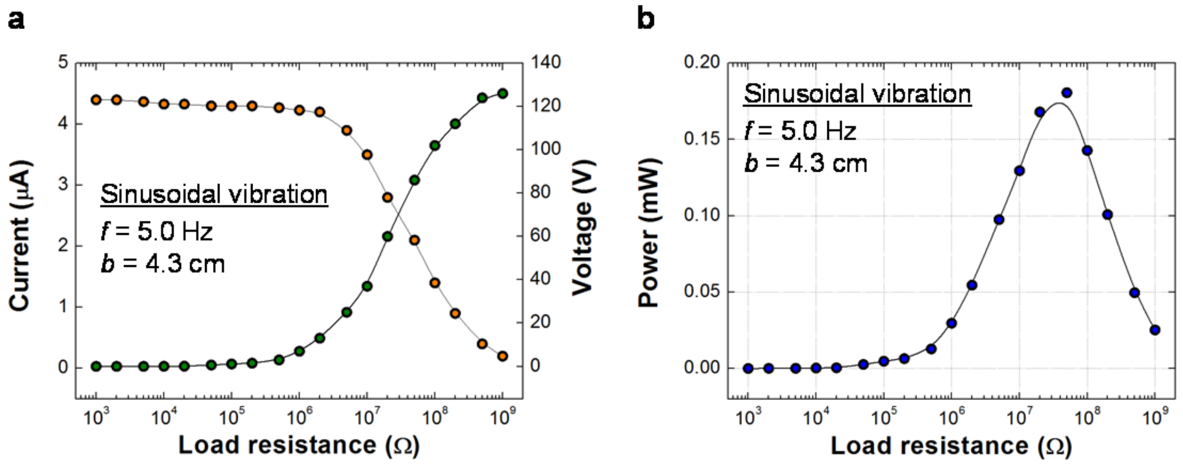

Figure S3. Output voltage, current, and power under various load resistances

Measurement Setup for Humidity Analysis

Figure S4 describes measurement apparatus for analysis of humidity-dependent electrical characteristics. The FO-TEG is placed inside of a closed acryl chamber. The humidity inside the acryl chamber is controlled by a humidifier and a humidity sensor. Vibration is applied by the electrodynamic shaker (LW-140-110, Labworks), which amplitude and frequency are controlled by a function generator (33120, hp) and power amplifier (pa-141, Labworks). The vibration generating equipments are placed outside of the acryl chamber, and only the vibrating head of the shaker is connected through a small hole of acryl chamber. Electrical signal of the FO-TEG is measured by the electrometer (Keithley 6514) and related LabVIEW program.

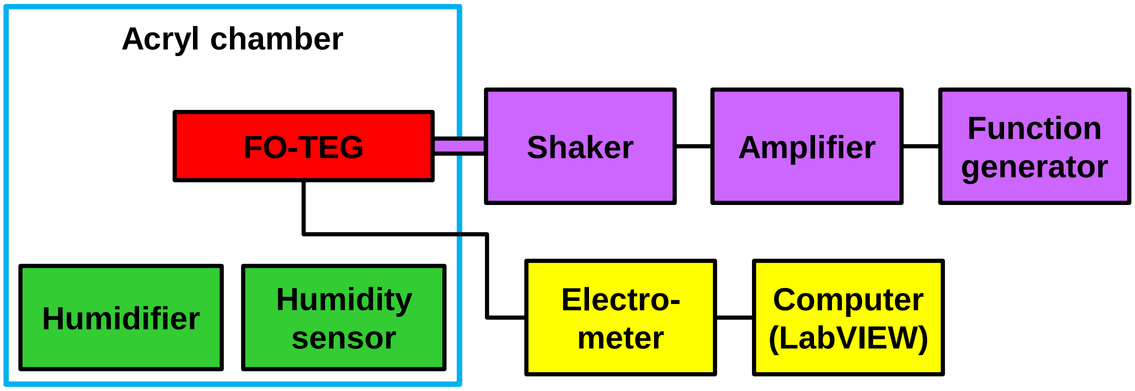

Figure S4. Measurement apparatus of FO-TEG

Characterization of Robustness

Figure S5 presents output voltage spectra before and after the intentionally applied stress. The measurement is conducted under the resonance oscillation, which produces the harshest stress to the contact interface. After the 18 hours of continuous measurement, which are corresponding to 324,000 cycles, output voltage degrades 9 to 11 %. Under nominal operations without harsh resonance oscillations, there is no perceivable degradation. The robustness of the FO-TEG can be considered within an acceptable range in most of practical applications. The observed degradation after the stress possibly stems from the damage of the interfacial nanostructure of PTFE. If a user wants to minimize the time-dependent degradation, shortening of nanowire length or rolling electrification design can be adopted (ref. [16] in the main paper).

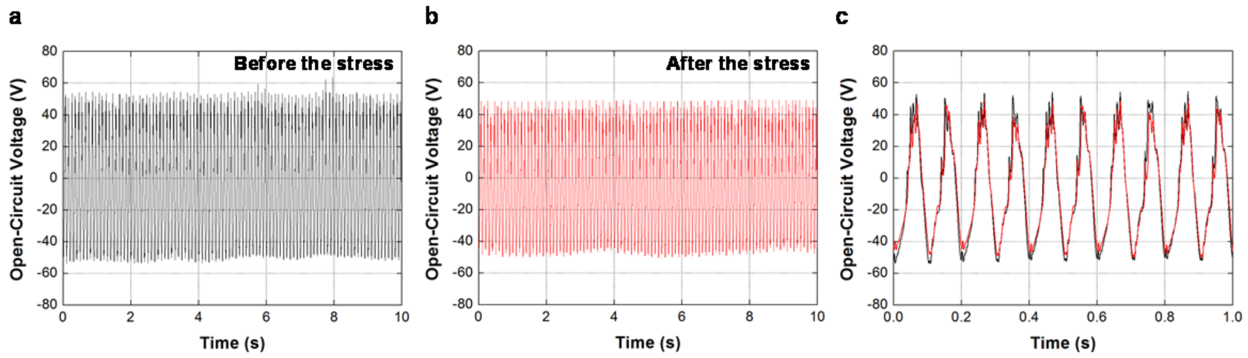

Figure S5. (a) Output voltage spectrum at the initial pristine state. (b) Output voltage spectrum after 18 hours of stress. (c) Magnified voltage spectra at the initial period (black) and after the stress (red).

Design Derivatives of FO-TEG

To further confirm the design flexibility, size scaling of the FO-TEG was conducted. Two FO-TEGs with reduced sizes were fabricated (Figure S6a-b), and conceptual applications of customized FO-TEGs are illustrated below (Figure S6c)

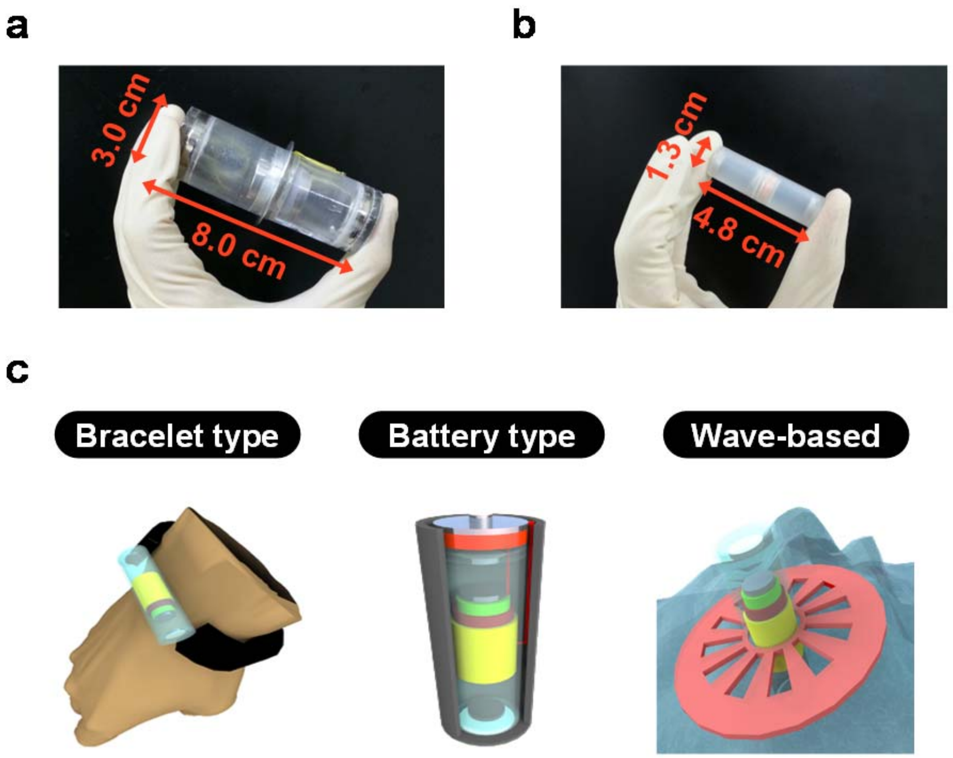

Figure S6. (a) A scaled FO-TEG with shortened tube length and cylindrical shape. (b) A further scaled FO-TEG. (c) Possible derivatives of FO-TEG for various applications.

A medium-scale FO-TEG based on a cylindrical tube was first fabricated (Figure S6a). The cylindrical structure is advantageous in terms of fabrication complexity, but accurate control of the diameter of the cylinder is required. The size of the FO-TEG can be further reduced for

possible handheld applications (Figure S6b). In this case, acryl is not a desirable material due to possible cracking, so an alternative for the plastic material is required. Conceptually, the shape and size of the FO-TEG can be further flexibly engineered depending on the specific application. Figure S6c suggests three possible applications which can be further realized using the customized FO-TEG. The energy managing circuit and charging component are omitted in the schematics. The bracelet type FO-TEG can harvest hand swinging motion while walking or running in daily life. The battery-type FO-TEG can serve as self-chargeable battery that is also highly compatible with a conventional battery socket. The wave-based FO-TEG can convert the sinusoidal motion of water waves into electric energy to power wireless sensor networks in the ocean.

Video Legends

Three videos are attached as supplementary information files.

Video S1. Measurement of FO-TEG in extremely humid environment

Video S2. Operation of LED array with electrodynamic shaker

Video S3. Operation of LED array with human running motion
